# Genomic, transcriptomic and RNA editing analysis of human MM1 and VV2 sporadic Creutzfeldt-Jakob disease

**DOI:** 10.1186/s40478-022-01483-9

**Published:** 2022-12-14

**Authors:** Martina Tarozzi, Simone Baiardi, Claudia Sala, Anna Bartoletti-Stella, Piero Parchi, Sabina Capellari, Gastone Castellani

**Affiliations:** 1 Department of Experimental, Diagnostic and Specialty Medicine, University of Bologna, 40139 Bologna, Italy; 2 Programma di Neuropatologia delle Malattie, Neurodegenerative, IRCCS Istituto delle Scienze Neurologiche di Bologna, 40139 Bologna, Italy; 3 Department of Biomedical and Neuromotor Sciences, University of Bologna, 40139 Bologna, Italy

**Keywords:** Prion diseases, Sporadic Creutzfeldt-Jakob disease, NGS, RNA sequencing, Phenotypic heterogeneity, Prion strains, Multi-omics analysis, RNA editing, Genetic modifiers

## Abstract

Creutzfeldt-Jakob disease (CJD) is characterized by a broad phenotypic spectrum regarding symptoms, progression, and molecular features. Current sporadic CJD (sCJD) classification recognizes six main clinical-pathological phenotypes. This work investigates the molecular basis of the phenotypic heterogeneity of prion diseases through a multi-omics analysis of the two most common sCJD subtypes: MM1 and VV2. We performed DNA target sequencing on 118 genes on a cohort of 48 CJD patients and full exome RNA sequencing on post-mortem frontal cortex tissue on a subset of this cohort. DNA target sequencing identified multiple potential genetic contributors to the disease onset and phenotype, both in terms of coding, damaging-predicted variants, and enriched groups of SNPs in the whole cohort and the two subtypes. The results highlight a different functional impairment, with VV2 associated with higher impairment of the pathways related to dopamine secretion, regulation of calcium release and GABA signaling, showing some similarities with Parkinson’s disease both on a genomic and a transcriptomic level. MM1 showed a gene expression profile with several traits shared with different neurodegenerative, without an apparent distinctive characteristic or similarities with a specific disease. In addition, integrating genomic and transcriptomic data led to the discovery of several sites of ADAR-mediated RNA editing events, confirming and expanding previous findings in animal models. On the transcriptomic level, this work represents the first application of RNA sequencing on CJD human brain samples. Here, a good clusterization of the transcriptomic profiles of the two subtypes was achieved, together with the finding of several differently impaired pathways between the two subtypes. The results add to the understanding of the molecular features associated with sporadic CJD and its most common subtypes, revealing strain-specific genetic signatures and functional similarities between VV2 and Parkinson’s disease and providing preliminary evidence of RNA editing modifications in human sCJD.

**Supplementary Information:**

The online version contains supplementary material available at 10.1186/s40478-022-01483-9.

## Background

Prion diseases are rare, invariably lethal and rapidly progressive neurodegenerative disorders that affect humans and other species of mammals [1]. Sporadic Creutzfeldt-Jakob disease (sCJD) is the most common and best studied human prion disease. It is characterized by a wide phenotypic spectrum regarding first symptoms, disease progression, and histo-molecular features. Current sCJD classification recognizes six main clinical and pathological phenotypes that largely correlate at the molecular level with the genotype at *PRNP* codon 129 (methionine, M, or valine, V) and the protein type (1 or 2) [2] accumulated in the brain. Among the six sCJD subtypes, MM1 and VV2 are the most common: MM1 accounts for ~ 65% of cases, and it is characterized by a rapid disease progression that, on average, has an age of onset of 70 years and a clinical disease duration of 4 months [3, 4]. The VV2 phenotype accounts for 15–20% of sCJD cases and is associated with a slightly longer disease (6 months on average) that appears earlier (64.5 years on average). While rapidly progressive dementia is the main clinical feature of MM1 phenotype, VV2 presents with prominent cerebellar and subcortical impairment [3, 5, 6]. Noteworthy, these subtypes not only show a difference in the clinicopathological phenotype, but transmission studies of the disease in syngeneic hosts demonstrated that sCJD MM1 and VV2 behave biologically as different prion strains, namely the M1 and V2 [7–9].

Recent research aimed mainly at a better understanding of the genetic risk factors and modifiers associated with the onset and phenotypic expression of the sporadic disease: Genome-Wide Association Studies (GWAS) of large sCJD cohorts confirmed the significant association with *PRNP* codon 129, the strongest genetic risk factor [10]. This work also identified two other loci associated with an increased risk of sCJD, in *STX6 (*rs3747957) and *GAL3ST1* (rs2267161) genes, indicating intracellular trafficking and sphingolipid metabolism as probable triggering mechanisms and corroborating the likely shared molecular dysregulation with other prion-like disorders [10]. In terms of gene expression, microarray and RNA sequencing have been applied to determine the most affected biological processes and molecular pathways at various disease stages. Most of the available knowledge comes from murine models: according to current literature [11–13], in the early disease stage, prominent changes in gene expression affect immune response through the complement system associated with microglia and astrocyte activation. During the intermediate stages of PrP^Sc^ accumulation, the transcriptional profile seems to alter pathways involving membrane regulation and vesicle traffic, with the activation of sphingolipid, glycosaminoglycans, and cholesterol metabolisms. In the final stage, a transcriptional down-regulation of genes associated with synaptic transmission and axonal growth occurs, followed by activation of cellular processes associated with apoptosis. Only a few studies about gene expression changes in human samples exist [14–17]. Although these studies provide information only about the final stage of the disease, they gain the advantage of focusing on a bona fide sporadic disease, differently from animal models that can describe only the acquired forms. These works highlight a prominent impairment of gene expression profiles that seem to parallel processes observed in animal models.

Further studies are needed to understand better the molecular mechanisms undergoing the sporadic disease and the biological pathways associated with its different phenotypes. This last aspect is crucial since the strain phenomenon, first described in prion diseases, has now been expanded to other more prevalent proteinopathies, like Parkinson's and Alzheimer's Diseases [18]. In this work, we performed DNA target sequencing on 118 genes in a cohort of 48 MM1 and VV2 sCJD, and RNA sequencing on postmortem brain samples (frontal cortex) in a subset of 16 cases (8 MM1, 8 VV2). On the genomic layer, data science and statistical analysis identified putative genetic modifiers and recurrent genetic patterns in the two classes, while RNA sequencing characterized differentially expressed genes and enriched pathways in the two subtypes/strains. Finally, the integration of these two omics layers also provided proof of RNA editing events in human disease, confirming the previous finding in murine models [19]. These results add to the current understanding of the molecular biology underlying prion diseases and the strain phenomenon.

## Results

### DNA target sequencing: putative genetic modifiers and recurrent genetic patterns

We analyzed DNAs of 48 Italian patients diagnosed with definite sporadic CJD MM1 and VV2 (Additional file 1: Table S1), with the Neurodegeneration (Illumina) gene panel (Additional file 1: Table S8). After applying filters described in materials and methods, 57,005 different variants were identified in the 118 analyzed genes. We observed 42,169 different variants in the MM1 group and 47,594 in the VV2. The numerical distribution of variants in each gene showed no significant difference between the two sCJD subtypes. On average, each sample carried 14,369 variants (σ = 823). Also, in this case, we found no significant difference in the average number of variants, even though MM1 samples showed a higher homogeneity compared to VV2 samples (MM1: 14,366 σ = 528, VV2: 14,373 σ = 1051).

### Variants in VV2 and MM1 affect different genes

**Fig. 1:**
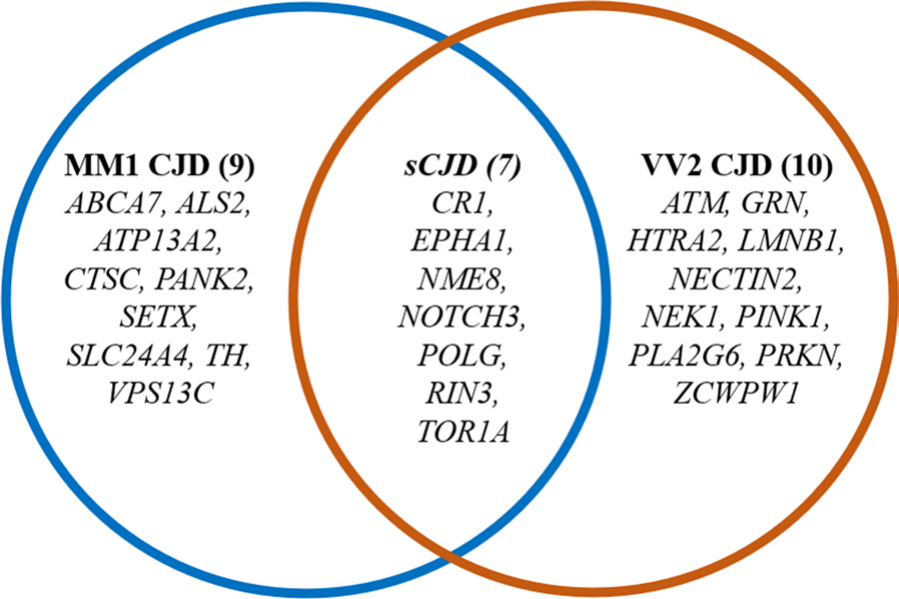
Venn diagram illustrating the distribution of gene harboring at least one likely pathogenic variant in the different groups, obtained from DNA target sequencing

Variants of interest were selected based on their consequence and predicted effect. Thirty-five different missense variants were predicted as “probably/likely pathogenic” or “deleterious” by both Polyphen2 and SIFT predictors. These variants, reported in Table 1, involve 26 genes. Seven variants were found more than once in the dataset, for a total of 62 findings of likely deleterious variants, equally distributed between the two subtypes (31 in MM1 and 31 in VV2). No difference was found in the number of samples carrying at least one putative damaging mutation between the two subtypes (17 MM1 and 16 VV2). Of the 26 genes involved in these possibly damaging variants, seven are affected in both MM1 and VV2 patients (*CR1, EPHA1, NME8, NOTCH3, POLG, RIN3, TOR1A),* while nine genes were affected only in MM1 samples *(ABCA7, ALS2, ATP13A2, CTSC, PANK2, SETX, SLC24A4, TH, VPS13C*) and ten genes only in VV2 samples (*ATM, GRN, HTRA2, LMNB1, NECTIN2, NEK1, PINK1, PLA2G6, PRKN, ZCWPW1*). Therefore, even though the number of likely pathogenic variants shows no difference between subtypes, specific genes seem to be affected exclusively in one subtypes and not in the other (Fig. 1).

**Table 1:** List of 35 probably damaging missense variants identified in this sporadic cohort, result of DNA target sequencing

VID	Gene	HGVSC	HGVSP	SIFT Prediction	PolyPhen2 prediction	Clinvar annotation
19:1046239:G	*ABCA7*	c.1456C > G	p.(Pro486Ala)	Deleterious	Probably damaging	NA
19:1047336:A	*ABCA7*	c.2026G > A	p.(Ala676Thr)	Deleterious	Probably damaging	Benign [single provider]
19:1058635:T	*ABCA7*	c.5168C > T	p.(Ser1723Leu)	Deleterious	Probably damaging	NA
2:202587783:T	*ALS2*	c.3685T > A	p.(Trp1229Arg)	Deleterious	Possibly damaging	NA
11:108117787:T	*ATM*	c.998C > T	p.(Ser333Phe)	Deleterious	Probably damaging	Conflicting interpretations: Uncertain significance(1); Benign(2); Likely benign(12)
1:17312787:A	*ATP13A2*	c.3472C > T	p.(Arg1158Cys)	Deleterious​	Possibly damaging​	Uncertain significance​
1:207680070:T	*CR1*	c.313C > T	p.(Arg105Cys)​	Deleterious​	Probably damaging​	NA
1:207739203:T	*CR1*	c.2537C > T​	p.(Ser846Phe)​	Deleterious​	possibly damaging​	NA
11:88027209:C	*CTSC*	c.1357A > G​	p.(Ile453Val)​	Deleterious​	possibly damaging​	Benign/Likely benign​
7:143088584:T​	*EPHA1​*	c.2897G > A​	p.(Arg966His)​	Deleterious​	Probably damaging​	NA
7:143092269:A​	*EPHA1​*	c.2090C > T​	p.(Pro697Leu)​	Deleterious​	Possibly damaging​	NA
17:42427095:A​	*GRN​*	c.325G > A​	p.(Gly109Arg)​	Deleterious​	Probably damaging​	Uncertain significance
2:74759825:A​	*HTRA2​*	c.1195G > A​	p.(Gly399Ser)​	Deleterious​	Probably damaging​	Benign/Likely benign
5:126158560:T​	*LMNB1​*	c.1474G > T​	p.(Ala492Ser)​	Deleterious​	possibly Damaging​	NA
19:45375208:T​	*NECTIN2​*	c.577C > T​	p.(Arg193Trp)​	Deleterious​	Probably damaging​	NA
4:170398474:C​	*NEK1​*	c.2235T > G​	p.(Asn745Lys)​	Deleterious​	Probably damaging​	Conflicting interpretations: Likely pathogenic(1); Benign(3); Likely benign(2)
7:37923923:C​	*NME8​*	c.1013T > C​	p.(Ile338Thr)​	Deleterious​	Probably damaging​	Benign
7:37924854:A​	*NME8​*	c.1247G > A​	p.(Ser416Asn)​	Deleterious​	Probably damaging​	NA
7:37936557:A​	*NME8​*	c.1630G > A​	p.(Ala544Thr)​	Deleterious​	Probably damaging​	Benign/Likely benign​
19:15273335:T​	*NOTCH3​*	c.5854G > A​	p.(Val1952Met)​	Deleterious​	Probably damaging​	Benign/Likely benign​
19:15289863:A​	*NOTCH3​*	c.3691C > T​	p.(Arg1231Cys)​	Deleterious​	Possibly damaging​	Conflicting interpretations: Pathogenic(2); Uncertain significance(5)
19:15290917:A​	*NOTCH3​*	c.3293C > T​	p.(Thr1098Ile)​	Deleterious​	Possibly damaging​	NA
20:3888719:A​	*PANK2​*	c.775G > A​	p.(Gly259Arg)​	Deleterious​	Probably Damaging​	NA
1:20976976:A​	*PINK1​*	c.1538G > A​	p.(Gly513Asp)​	Deleterious​	Probably damaging​	NA
22:38539240:A​	*PLA2G6​*	c.481C > T​	p.(Arg161Cys)​	Deleterious​	Probably damaging​	Uncertain significance​
15:89865073:C​	*POLG​*	c.2492A > G​	p.(Tyr831Cys)​	Deleterious​	Possibly damaging​	Benign/Likely benign​
6:161781201:A​	*PRKN​*	c.1204C > T​	p.(Arg402Cys)​	Deleterious​	Probably damaging​	Conflicting interpretations: Uncertain significance(2); Benign(1)
14:93119136:A​	*RIN3​*	c.1742G > A​	p.(Arg581Gln)​	Deleterious​	Probably damaging​	NA
14:93142861:C​	*RIN3​*	c.2377T > C​	p.(Tyr793His)​	Deleterious​	Possibly damaging​	NA
9:135202325:C​	*SETX​*	c.4660T > G​	p.(Cys1554Gly)​	Deleterious​	Probably damaging​	Benign/Likely benign​
14:92905737:C​	*SLC24A4​*	c.377T > C​	p.(Leu126Pro)​	Deleterious​	Probably damaging​	NA
11:2189817:C​	*TH​*	c.484T > G​	p.(Phe162Val)​	Deleterious​	Possibly damaging​	Uncertain significance​
9:132580901:G​	*TOR1A*	c.646G > C	p.(Asp216His)	Deleterious	Possibly damaging	Benign
15:62269347:C​	*VPS13C*	c.2342T > G	p.(Leu781Trp)	Deleterious	Probably damaging	NA
7:100016781:C​	*ZCWPW1*	c.314A > G	p.(Glu105Gly)	Deleterious	Probably damaging	NA

### Allele frequencies differ between SCJD patients and the European population but not between VV2 and MM1

Allele frequencies were compared between the two considered subtypesand between the whole sCJD cohort and the European population. The comparison between MM1 and VV2 subtypes did not provide any statistically significant result except for codon 129 and three other SNV in its proximity, which was expected given the experimental design. The comparison between sCJD patients versus allele frequencies of the European population reported in the GnomAD database highlighted 237 variants with a statistically significantly different allele frequency (p-adj < 0.05). Of them, 71 are non-intronic (missense, synonymous, 3′/5′ UTR, splice region variants) and distributed in 36 different genes. Functional analysis with over representation methods on these 36 genes (Additional file 1: Table S2) showed an enrichment of several biological processes, many of which involved in synaptic regulation, chaperon binding and regulation of cell death, and a strong association with neurodegenerative processes. Three of the overrepresented variants in the sCJD cohort were also predicted as probably pathogenic by SIFT and PolyPhen2 variant predictors: *GRN* p.Gly109Arg (p.adj = 0.02, sample #17, VV2), *NME8* p.Ser416Asn (p.adj = 0.03, sample #40, MM1) and *RIN3* Arg581Gln (p.adj = 0.03, sample #8, VV2). For none of these three variants is available a clinical significance value on ClinVar. Each one of these variants was found in only one sample in the analyzed cohort, thus, a validation in a larger cohort would be necessary to confirm this preliminary finding.

### Unsupervised clustering of variants highlights patients’ stratification

**Fig. 2:**
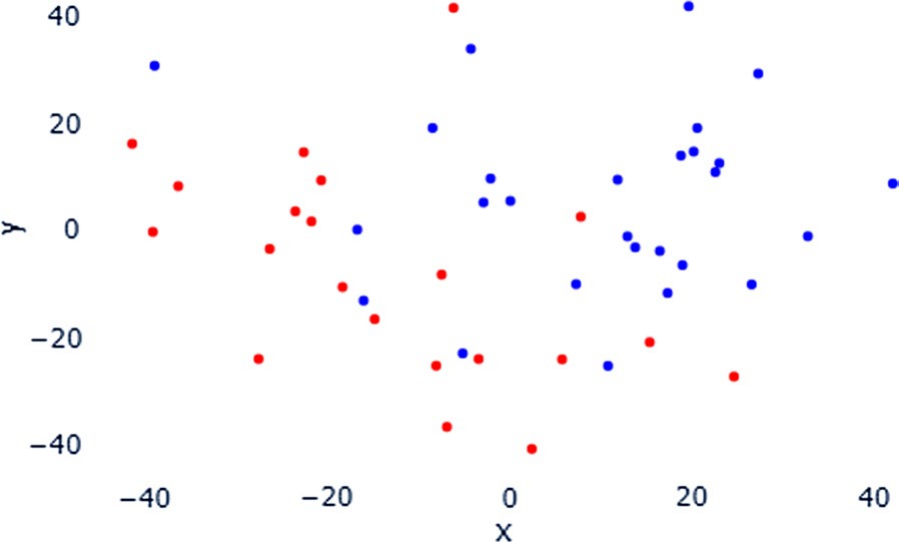
PCA plot of the genetic mutational profile derived from DNA target sequencing. In the plot, each point represents a sample, colored accordingly with the K-Means clustering. K-Means recognizes two clusters mainly distributed along the first principal component of the PCA plot

Principal Component Analysis (PCA) of the whole genetic mutational profile (including 57,005 variants) in this dataset is reported in Fig. 1, where quite a sparse distribution of the 48 sample is visible. The first eighteen principal components explain 50% of the overall variance of the dataset. The main contributor of PC1 is a variant in the genomic region of the gene *PARKN* (chr6-163069504-G-A), while PC2’s main contributor is a SNV in the genomic region of the gene *PICALM* (chr11-85710180-G-A). K-means clustering based on PC1 and PC2 identified two numerically similar classes, separating samples along PC1 (Fig. 2). None of the available technical, biological or clinical features, as well as other known sources of variation like sex and geographical origin of the patient, matched the clusterization. Taken together these two findings suggest an overall homogenous genetic background between the two subtypes, as also depicted by the allele frequencies analysis between subtypes. Despite this, a clusterization based mainly on variants in the *PARKN* genomic region is observed, that anyway does not match any of the most important phenotypic features or confounders.

### Intronic variants in PRNP and FERMT2 discriminate MM1 and VV2 patients

**Fig. 3:**
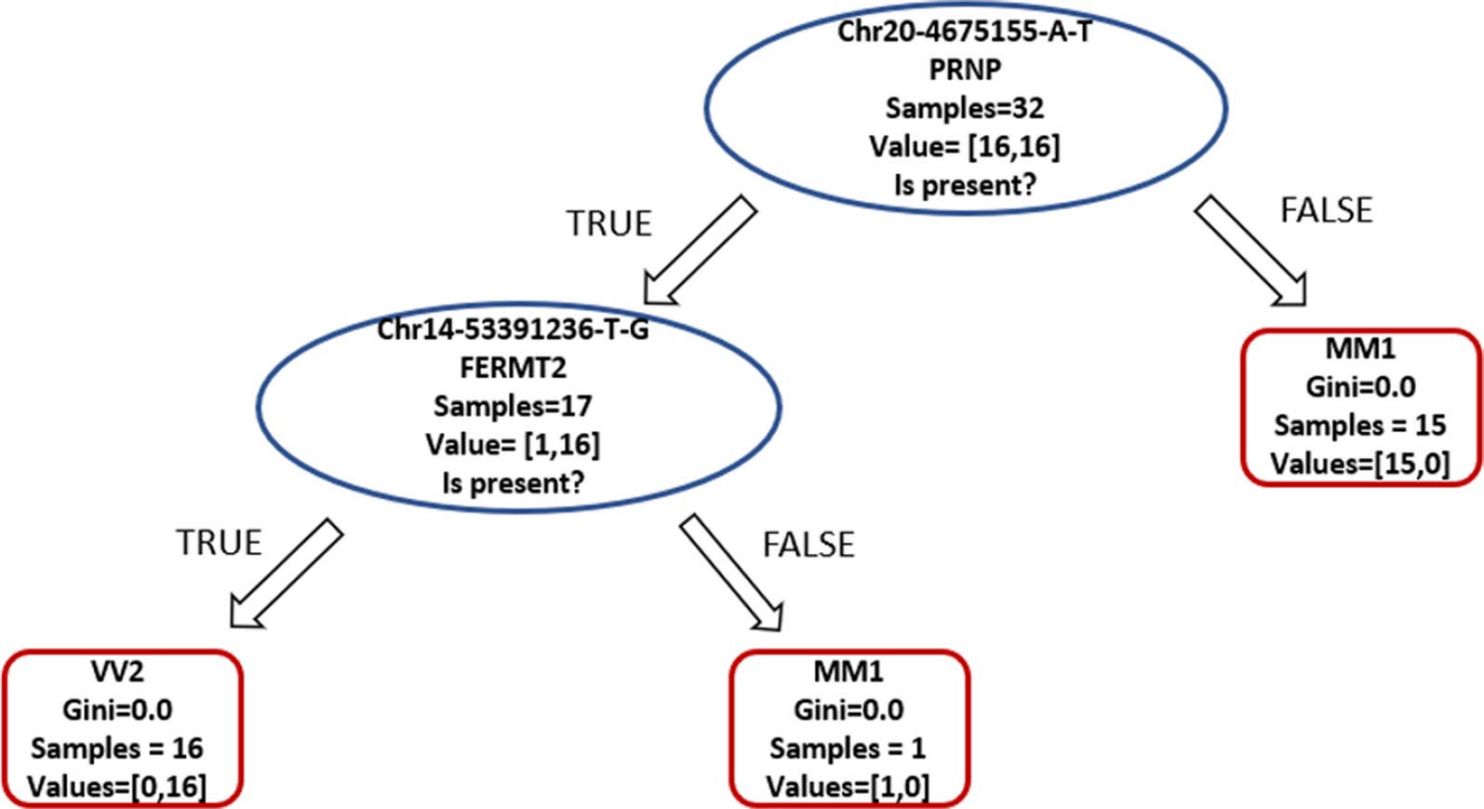
Decision tree graphical representation, resulted from the supervised classification of 57,004 variants identified in this dataset from DNA target sequencing. The classification was performed using two variants as parameters. First, a sample did not carry the SNV A/T in position 4,675,155 of the *PRNP* gene, it was assigned to the MM1 class. Afterwards, if a sample carried that variant and also the SNV T/G in position 53,391,236 in the *FERMT2*, it was assigned to the class VV2. Classification metrics MM1: Precision = 0.73, Recall = 1, F1 = 0.84. Classification metrics VV2: Precision = 1, Recall = 0.62, F1 = 0.77

Supervised classifiers were used for automatic recognition of genetic patterns among the 57,005 variants identified in this dataset. Decision trees were selected as classifiers due to their previous employment in clinical genomics and precision medicine applications to interpret the role of genetic variants in complex diseases [20, 21]. The classification was achieved perfectly, with 100% accuracy on the test set, based on the codon 129 (chr20-4680251-A/G). To test for other recurrent genetic patterns that could characterize the two phenotypic groups, we removed from the input data the variant corresponding to the codon 129. As expected, accuracy decreased both in the training set and in the test set, but interestingly the classifier managed to distinguish the two diseases with good accuracy (training = 1, test 0.81). The classification is based on two intronic variants, in *PRNP* and *FERMT2* genes (Fig. 3). According to common databases and genomic search engines such as VarSome [22], OMIM [23], ClinVar [24] or HGMD [25], the intronic variant in *FERMT2* was never previously reported as a functional intronic variant. The intronic variant in *PRNP* is also referred to with the ID rs6037932, this variant was used in a phylogenetic study about founder effect in another prion disease (FFI) in 2008 [26]. This SNP is in the intronic region between exon 1 and 2, 5 kb away from codon 129. This SNP is not in complete linkage disequilibrium with the allele 129 V, even though in this cohort and in the previously cited work, it is more frequently associated with Valine. On the other hand, the *FERMT2* variant Chr14-53391236-T-G in this cohort is always associated with the allele 129 V.

### VV2 and MM1 patients show characteristic gene expression profiles

**Fig. 4:**
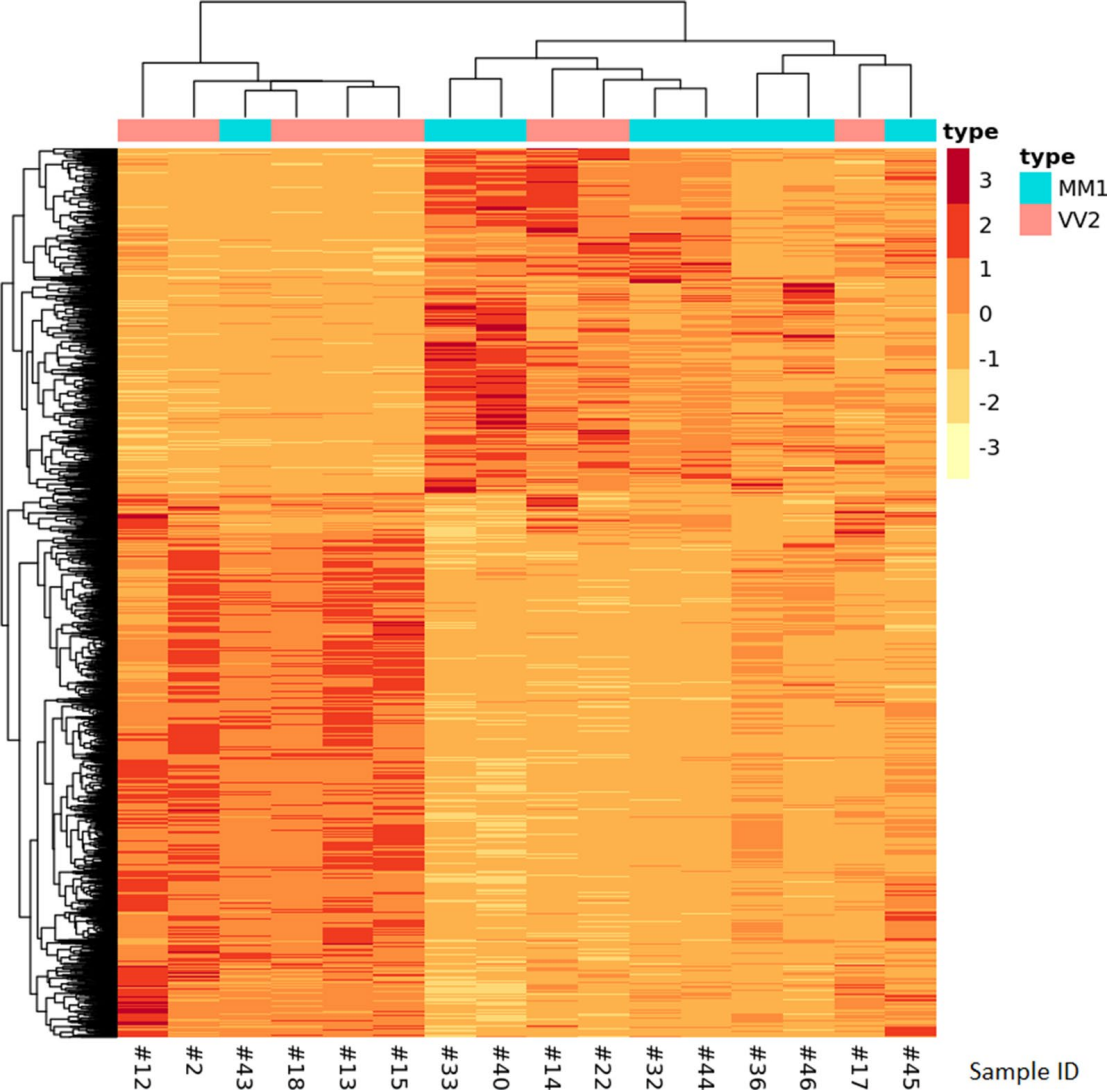
Heatmap of the 1798 differentially expressed genes in VV2 subtype compared to MM1, result of the transcriptomic analysis. Hierarchical clustering identified two clusters, one enriched in MM1 samples (identified by a light blue label) and the other with VV2 more represented (pink label)

From DGE analysis, 1696 differentially expressed protein coding transcripts were identified in the comparison between VV2 and MM1 (Fig. 4). Among them, 1153 transcripts are significantly over expressed in VV2 compared to MM1 (518 with Log Fold Change (LFC) > 1, Additional file 1: Table S6), while 543 are significantly under over expressed in MM1 compared to VV2 (209 with LFC < -1, Additional file 1: Table S7). Differential expression of the six genes with the most extreme LFC values was validated with digital droplet PCR (ddPCR) (Additional File 1, top 10 Differnetially Expressed Genes (DEG) per subtypeand ddPCR), confirming the trend observed in RNAseq. The heatmap of the expression profiles of differentially expressed genes reported in Fig. 4 shows a good clusterization of the two subtypes based on the results of the differential gene expression analysis: two clusters are identified, a smaller one made of five VV2 samples and one MM1 sample, and a larger cluster made of ten samples, seven of which are MM1 and three are VV2.

### Functional enrichment analysis shows impairment of pivotal pathways for SCJD pathology

**Fig. 5:**
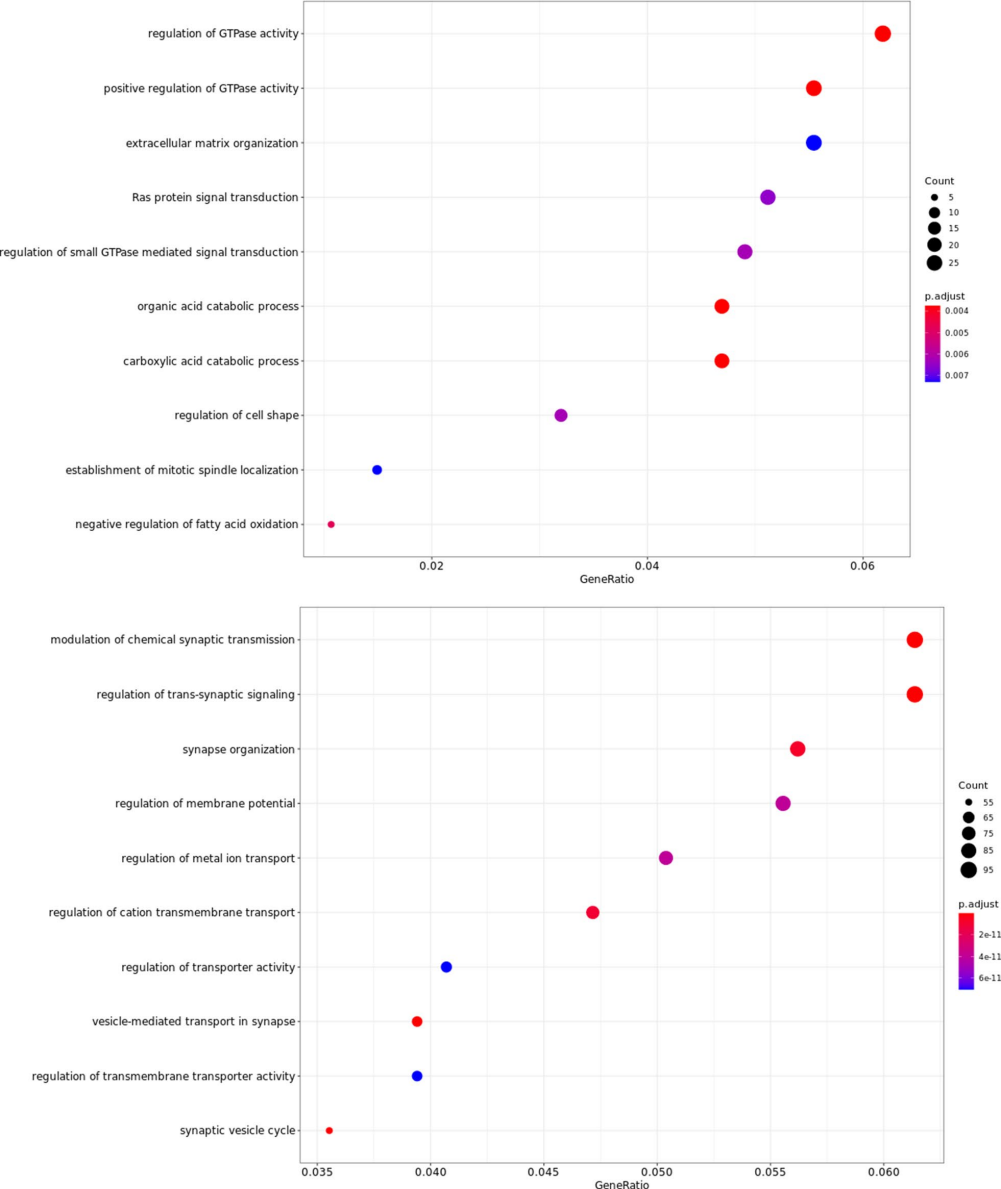
Dot plot of the top 10 enriched pathways in the MM1 subtype (upper panel) and in the VV2 subtype (bottom panel) compared to the other, derived from over representation analysis of DEGs obtained with RNAseq

To gain functional insights about the consequences of differentially expressed genes, functional enrichment was performed with three different computational methods: over representation analysis, gene set enrichment analysis and protein–protein interaction networks. Over representation analysis of MM1 overexpressed genes, identified 21 pathways with a significant enrichment: the most affected pathways were regulatory pathways mediated by guanosine triphosphatases (GTPases), regulation of catabolic processes and maintenance of proper cell morphology and matrix organization (Fig. 5, Additional file 1: Table S5).

**Fig. 6:**
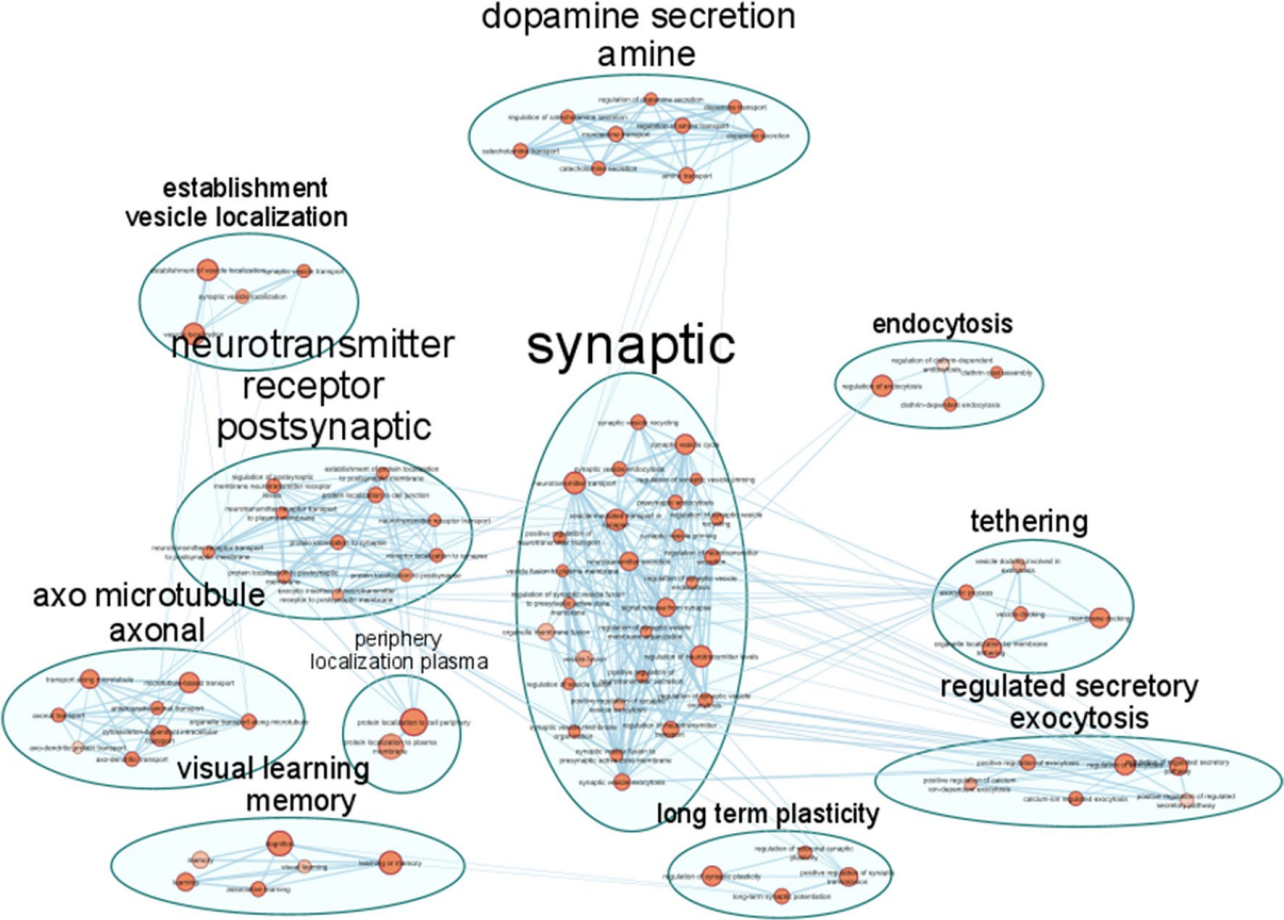
Region of the network representing enriched pathways grouped by non-redundant functional modules in the VV2 group, result of transcriptomics analysis. Most of the biological processes can be grouped into the functional module of synaptic regulation and vesicle trafficking. Notably, in the upper part of the network a cluster representing pathways involved in dopamine secretion is represented. See Table 2 for the complete number of clusters and nodes

In the VV2 group, based on the list of 1153 significantly overexpressed genes, 634 biological processes were significantly enriched, with several pathways referring to synaptic regulation and vesicle trafficking associated with the strongest enrichment (Fig. 5). To highlight the functional modules starting from hundreds of interconnected and overlapping pathways, Cytoscape was used to summarize non redundant functional modules and visualize interconnections between pathways. As summarized in Fig. 6 and Table 2, pathways involved in synaptic regulation and regulation of endo- and exo-cytosis are not only associated with the lowest p values, but they also represent the most numerous classes of pathways grouped in the functional modules plotted in the network.

**Table 2:** Non-redundant functional modules grouping pathways showing a significant enrichment based on the overexpressed genes in VV2, derived from transcriptomics experiments

Cluster	Nodes	Cluster	Nodes
Vesicle fusion synaptic	28	Regulation dendrite morphogenesis	4
Activity transmembrane transporter	15	Response metal substance	4
Assembly synapse pre-synapse	12	Central nervous neuron	3
Muscle contraction cardiac	11	Chemical postsynaptic excitatory	3
Neurotransmitter receptor postsynaptic	11	Regulation depolarization potential	3
Dopamine secretion amine	9	Amino acid starvation	2
Membrane mitochondrial permeability	9	Peptide hormone insulin	2
Microtubule-mediated axonal transport	8	Periphery localization plasma	2
Anion chloride transmembrane	7	RAC signal transduction	2
Cellular response stimulus	7	Regulation macro autophagy	2
Regulation ph reduction	7	Negative regulation cell	1
Developmental growth extension	6	Negative regulation microtubule	1
Regulated secretory exocytosis	6	Neurofilament cytoskeleton organization	1
Visual learning memory	6	Neuron apoptotic process	1
Adult walking behaviour	5	Neuron recognition	1
Cytosol sarcoplasmic reticulum	5	Post Golgi vesicle	1
Docking organelle tethering	5	Protein folding	1
Positive protein intracellular	5	Protein homo-oligomerization	1
Sodium ion transmembrane	5	Regulation dephosphorylation	1
Clathrin coat endocytosis	4	Regulation proteasomal protein	1
Dendritic spine organization	4	Response temperature stimulus	1
Establishment vesicle localization	4	Spontaneous synaptic transmission	1
Long term plasticity	4	Synaptic transmission gabaergic	1
Potassium ion transmembrane	4	Synaptic transmission glutamatergic	1

The table shows the complete number of clusters and nodes that are graphically represented in Fig. 6

Gene set enrichment analysis (GSEA) assumes that also weaker but coordinated changes in sets of functionally related genes can have significant effects, therefore in this type of functional analysis all transcripts are considered. Despite being based on different assumptions compared to the previous approach, these results reinforce the previous findings of increased regulation of synaptic functionality in the VV2 group compared to the MM1, both in structural terms affecting neuronal projection and axonogenesis, and in functional terms affecting synapse activity with altered regulation of mono and divalent cations transport. Lastly, Protein–Protein Interaction networks (PPIn) provide information on physical interactions of proteins encoded by differentially expressed genes. Here, only direct biophysical interactions were considered (i.e., molecular docking) mapped on STRING interactome and based on these interactions, pathway enrichment was performed on KEGG database. Also with this approach, synapse regulation results to be the most evident pathway differently affected in this comparison between VV2 and MM1 subtypes, and in the MM1 group highlighted an impairment of the regulation of nucleotide binding mechanisms, which is coherent with the previously described result of a positive regulation of GTPase activity.

### Integration of DNA and RNA sequencing data highlights RNA editing events

**Fig. 7:**
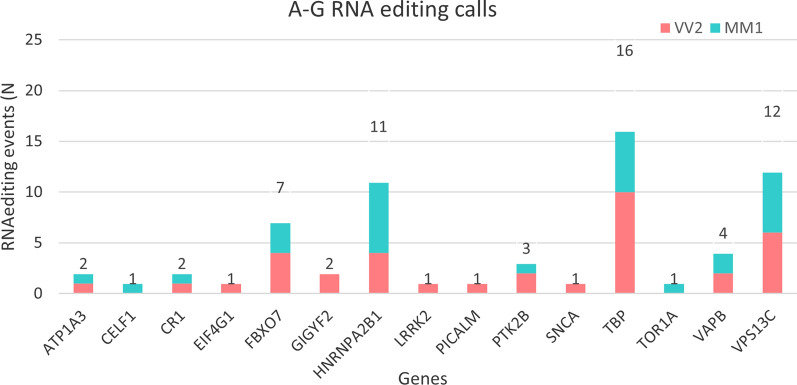
Bar plots reporting the number of RNA editing events per gene and CJD subtype distribution, derived from the integration of DNA and RNA sequencing data. The genes harboring the highest number of editing events are known players of CJD and/or other proteinopathies, like TATA-Box Binding Protein—*TBP* (20) and F-box only protein 7—*FBXO7* (16), Vacuolar protein sorting-associated protein 13C—*VPS13C* (12) and Heterogeneous nuclear ribonucleoproteins A2/B1—*HNRNPA2B1* (11)

In the genomic regions in which both DNA and RNA sequencing data were available, RNA editing events were analyzed. After applying the quality filters reported in materials and methods, 36 sites in the VV2 and 31 sites in MM1 corresponding to A-to-G changes were identified, consistent with ADAR-mediated RNA editing. The full table of all editing events is reported in Additional File 1: Table S3. We found 15 genes carrying at least one edited site, no significant differences between MM1 and VV2 CJD in terms of number of events and genes involved were observed (Fig. 7). As expected by previous works on RNA editing in aging and neurodegeneration [27], many of the editing events are annotated in regulatory regions such as splicing regions and 3′UTRs, even though also modifications in coding regions were found (Table 3). Interestingly, the genes harboring the highest number of editing events, *TBP* (16), *VPS13C* (12), *HNRNPA2B1* (11) and *FBXO7* (7), are known players of CJD and/or other proteinopathies*.* Functional enrichment analysis performed on these 15 genes highlighted a significant (p.adj < 0.05) involvement of relevant cellular components and biological processes in the disease, such as neuronal cell body and distal axon (GO:0043025, GO:0150034) and synaptic vesicle transport and localization (GO:0048489, GO:0097479) (Additional file 1: Table S4).

**Table 3:** List of all the putative RNA editing modifications observed in the cohort of 16 samples, in the 118 genes and regulatory regions covered by both DNA target sequencing and RNA sequencing

Region	Position	Consequence	Class_counts	Gene
Chr19	42,490,154	Splicing, intronic	1 MM1, 1 VV2	*ATP1A3*
Chr11	47,493,737	Splicing, intronic	1 MM1	*CELF1*
Chr1	207,741,245	Synonimous	1 VV2	*CR1*
Chr3	184,039,770	Synonimous	1 VV2	*EIF4G1*
Chr22	32,871,936	Intronic	3 MM1, 4 VV2	*FBXO7*
Chr2	233,712,272	Synonimous	2 VV2	*GIGYF2*
Chr7	26,236,175	Splicing, intronic	4 MM1, 2 VV2	*HNRNPA2B1*
Chr7	26,236,172	Splicing, intronic	3 MM1, 2 VV2	*HNRNPA2B1*
Chr12	40,671,989	Missense (Ile723Val)	1 VV2	*LRRK2*
Chr11	85,742,663	Splicing, intronic	1 VV2	*PICALM*
Chr8	27,255,263	Synonimous	1 MM1, 2 VV2	*PTK2B*
Chr4	90,749,343	Splicing, intronic	1 VV2	*SNCA*
Chr6	170,871,040	Synonimous	2 MM1, 4 VV2	*TBP*
Chr6	170,871,046	Intronic	2 MM1, 4 VV2	*TBP*
Chr6	170,871,013	Synonimous	2 MM1, 2 VV2	*TBP*
Chr9	132,576,302	Synonimous	1 MM1	*TOR1A*
Chr20	57,022,720	3′UTR	2 VV2 e 2 MM1	*VAPB*
Chr15	62,306,194	Splicing, intronic	6 MM1, 6 VV2	*VPS13C*

## Discussion

In this work, we investigated the issue of phenotypic heterogeneity in prion diseases by searching for molecular differences and similarities between the two most common sCJD subtypes, MM1, and VV2, that also represent the principal human prion strains (M1 and V2).

We found several shared features between the sCJD MM1 and VV2. At the genomic level, the two groups showed an overall similar genetic background, as demonstrated by the average number of SNV per sample, the statistical analysis of allele frequencies, and by the principal component analysis. Nevertheless, we also identified several variants that could act as risk factors or phenotypic modifiers. Our results suggest that sCJD could have polygenic contributions able to influence the prevalent strain, opening to the possibility to expand this hypothesis also to carriers of genetic forms of the disease in future studies. Indeed, the comparison between allele frequencies in the whole sCJD cohort and the healthy European population highlighted several variants with significantly different allele frequencies. Here, functional enrichment provided evidence of a probable downstream impairment of pivotal pathways for CJD pathology -such as chaperon binding, synaptic, and cell death regulation- suggesting a complex genetic background underlying the disease. This is also supported by the finding of probably pathogenic variants in other genes than *PRNP*. In this regard, we found meaningful differences between the two subtypes, with specific genes affected by probably pathogenic variants only in one subtype. In the MM1 subtype, we found variants in genes involved in a heterogeneous group of molecular processes and neurodegenerative diseases, while in the VV2, we found an over-representation of genes involved in PD, such as *HTRA2, PINK1,* and *PRKN* [28, 29]*. PINK1* exerts a protective role against mitochondrial dysfunction by activating mitochondrial quality controls mechanisms that mediate mitophagy and lysosomal function through phosphorylation of other mitochondrial proteins such as the E3 ubiquitin-protein ligase PRKN [30]. In addition, PINK1 is also involved in the mitochondrial unfolded protein response (UPRmt) through its interactions with HTRA2 [31]*.* Mitochondrial dysfunction has been associated with several neurodegenerative disorders, but very few studies are available in sCJD. Recently, Flønes et al. [32] showed a positive correlation between the level of impairment of the five respiratory complexes in neurons of both MM1 and VV2 CJDs with the severity of other neuropathological changes such as gliosis, vacuolation, and PrP^sc^ accumulation. The putative pathogenic variants related to PD are found only in the VV2subtype, suggesting a possible overlapping mechanism regarding mitochondrial quality control dysfunction in Parkinson disease and in VV2 sCJD.

At the transcriptomic level, a comparative analysis was carried out on post-mortem frontal cortex with RNA sequencing (cDNA capture). The similarity between VV2-CJD and PD was also evident at the transcriptomic level, where differential gene expression and functional analysis confirmed and expanded the finding of impairment of some key biological processes associated with PD, such as dopamine secretion, regulation of calcium release, GABA signaling, and mitochondrial permeability in sCJD VV2. This finding is supported by the high prevalence of parkinsonism and other movement disorders in prion diseases [33–37]. Specifically, VV2 and MV2 subtypes exhibit the most severe neuropathological changes, as defined by regional lesion profiles, in the midbrain (substantia nigra) and striatum within the spectrum of sCJD subtypes [2]. In particular, the midbrain and striatum are consistently affected from the earliest stages of the disease in sCJD VV2 [38]. As a result, within the first 2 months of disease onset, about 15% of patients have parkinsonian signs on neurological examination, rising to 35% considering the entire disease course [38].

We also observed in the VV2 subtype an upregulation of genes involved in synaptic regulation affecting both pre- and post-synaptic terminals. Similarly, VV2 showed overexpression of genes involved in vesicle transport and turnover compared to MM1. Both these biological processes have been described as strongly impaired in all CJD subtypes, especially in the mid and late disease stages [12, 14, 17]. Previous studies comparing CJD cases with controls reported that the intermediate phases of the disease are characterized by a substantial impairment of the transcriptional response directed to pathways involving vesicular trafficking, as well as activation of cholesterol synthesis and efflux, glycosaminoglycan metabolism, and sphingolipid synthesis and degradation [12]. Indeed, membrane composition and membrane microenvironment, in particular lipid rafts were the surface GPI-anchored PrP^C^ is enriched [39], is involved not only in the normal cellular processing and functions of PrP^C^ (i.e., localization, internalization, and intracellular trafficking) [40] but also in the conformational conversion of PrP^c^ into PrP^Sc^. In fact, evidence suggests that PrPSc can form pathogenic aggregated while bound to cellular membranes [41] and that PrP^Sc^ formation is most efficient when both PrP^C^ and PrP^Sc^ are anchored to contiguous membranes [42]. Additionally, it was shown that given their C-terminal attachment site, the GPI anchors twist along one side of the fibril while binding to membranes causing membrane distortions that are likely critical in disease pathogenesis and contribute to explaining the much faster course of CJD compared to other disorders [43].

The observed overexpression in VV2 compared to MM1 was concordant between all functional enrichment methods, suggesting that in the VV2subtype the downregulation of these processes may occur with less intensity. Previous works have already demonstrated that in CJD genes regulating these pathways are under-expressed compared to controls [14, 44]. In later disease stages, the impairment increases as genes associated with synaptic transmission and axon guidance are downregulated, and ultimately cellular processes related to cell death are activated [11, 12, 17].

In the MM1 group, the most affected pathways involved guanosine triphosphatases (GTPases)-mediated regulatory pathways, regulation of catabolic processes, maintenance of proper cell morphology and matrix organization. The small guanosine triphosphatases (GTPases) of the Ras superfamily are important regulators of key cellular processes such as cell cycle regulation, proliferation, intracellular trafficking, and apoptosis. Their involvement in neurodegenerative diseases is linked to many processes, particularly impairment of catabolic processes, vesicular trafficking, and regulation of apoptosis [45–47]. Notably, aberrant activity of GTPases and their regulators has been reported and studied extensively in the most common neurodegenerative disorders such as PD, ALS/FTD, and AD for its key role in synaptic maintenance and even as a possible therapeutic target in AD [48, 49]. The other pathway found to be differentially altered in sCJDMM1 is the dysregulation of catabolic process. This is another pathway that has been reported in all proteinopathies [45, 50]. Given their fundamental importance in the functional alteration of neurons and glial cells associated with the accumulation of misfolded proteins, these pathways are shared by several neurodegenerative diseases. Coherently with the genomic findings, MM1-CJD portrayed a gene expression profile where several biological processes shared by different neurodegenerative diseases were involved without an evident distinctive trait.

Interestingly, we found no significant differences between the two subtypes in terms of cell-types involved in immune response and neuroinflammation (see also cell type enrichment in Additional file 1: Table S9). According to the current literature [11, 12, 51], in the early stage of the disease, major changes in gene expression occur involving the immune response through the complement system and leukocyte infiltration, associated with the activation of microglia and astrocytes. Neuroinflammation and activation of the immune response are regulated from the earliest stages of the disease [51], therefore the lack of significant differences could be explained by the fact that in the terminal part of the disease these processes are already fully activated and widespread in both subtypes in a similar manner [52]. Lastly, the integration of genomic and transcriptomic data confirmed the previous findings of RNA editing events in CJD [19]*.* RNA editing is known to be involved in several neurodegenerative diseases [53, 54], and was described in animal models of CJD [19]. While our results confirm the presence of this type of epigenetic modification, none of the genes identified by Kanata and colleagues in the preclinical stages of the mouse model was found to be modified in the terminal stages of the human disease. The genes harboring the highest number of A-G editing events in our dataset were *TBP* and *FBXO7* (16), *VPS13C* (12), and *HNRNPA2B1*, which are strongly associated with other proteinopathies, such as PD or FTD/ALS. Accordingly, the functional enrichment analysis results on these genes again highlighted the involvement of pivotal pathways impaired in the disease, such as vesicle trafficking and protein quality control*.* RNA editing is still scarcely studied in prion diseases, even though it is the object of growing attention in other more common neurodegenerative disorders. All the described genes interested in RNA editing in our cohort are present in the annotation database of A-to-I RNA editing in AD (https://ccsm.uth.edu/Adeditome), [54] even though with different loci interested in A-G editing. These findings, together with previous works, highlight the presence of these post-transcriptional modifications in the brains of both human and animal models of CJD; therefore, further studies are needed to understand better the functional effects of these modifications in the disease. Our study is not free of limitations. In interpreting our results, the following should be considered: (1) the relatively small size of the cohorts analyzed; (2) the lack of proteomic data that prevents drawing conclusions about their downstream effects; and (3) given the focus on capturing differences between the two sCJD subtypes, which eliminates shared alterations and consequently impacts on the altered pathways identified, the impossibility of a comprehensive comparison with data sets from other neurodegenerative diseases. Further studies on RNA editing in prion diseases and neurodegeneration coupling genomic, transcriptomic, and proteomic data could clarify these open issues.

## Conclusions

In this work, we performed a multi-omics analysis of the two most common subtypes of sCJD in human tissue samples. We identified several putative genetic contributors to the disease onset and phenotype and profiled subtype-specific gene expression alterations, revealing some type-specific genetic signatures and functional similarities only between VV2 CJD and Parkinson’s disease. This multi-omics analysis also provided evidence of RNA editing modifications in the disease, confirming results previously obtained in CJD mouse models. These results show a complex misregulation involving alterations at genomic, transcriptomics, and epigenetic (RNA editing) levels in CJD. This work improves the understanding of the molecular pathology of the disease, highlighting novel putative players in the disease onset and its phenotypic heterogeneity, representing a step forward in the state-of-the-art in this field both from a biological and technological perspective.

## Methods

### Samples and experimental design

In this work, we performed DNA target sequencing on 118 genes in a cohort of 48 MM1 and VV2 sCJD, and, on a subset of 16 samples, RNA sequencing on postmortem brain. Forty-eight patients diagnosed with definite sporadic CJD, accordingly to the updated clinical diagnostic criteria for sporadic Creutzfeldt-Jakob disease [55], afferent to the IRCCS Institute of Neurological Sciences of Bologna were selected. We selected samples belonging to patients with genotype Met–Met (N = 24) or Val–Val (N = 24) at codon 129 of the *PRNP* gene, without any co-pathology, and with a clinically “pure” phenotype equally distributed between male and females (Additional file 1: Table S1). Ethical approval was obtained from the ethical board of our institution. For all subjects, written informed consent was provided. All methods were performed in accordance with the relevant guidelines and regulations.

### Next generation sequencing

Genomic DNA from peripheral blood or brain tissue was isolated using the Maxwell 16 extractor (Promega, Madison, WI, USA). DNA was quantified using the Quantus Fluorometer (Promega) with QuantiFluor double-stranded DNA system. DNA libraries were prepared with DNA Prep with Enrichment kit (Illumina, CA, USA) performing enrichment with Illumina Neurodegeneration panel (full gene list in Additional file 1: Table S8). We found this product the most appropriate for this work due to the combination between a standardized and field-specific targeted panel with the possibility to also cover also non-coding regulatory regions. Library prep was performed following instruction provided by the vendor. Paired end sequencing (150 × 150) was performed with a NextSeq 500 (Illumina, CA, USA).

On a subset of the previous forty-eight cohort, sixteen brain samples of sCJD patients with subtypes MM1 (N = 8) or VV2 (N = 8) were selected for RNA sequencing. All selected samples had tissue suitable for this analysis (body kept refrigerated (2–4 °C) before autopsy and with a post-mortem < 36 h to minimize RNA degradation) with mild to moderate lesions in the frontal cortex on histopathological examination. Total RNA was extracted from 50 mg of frozen frontal cortex with RNeasy Lipid Tissue Mini Kit (Qiagen) and quantified with NanoDrop 2000 (Thermo Scientific). Total RNA was subsequently treated with DNase I, RNase-free (Thermo Scientific). Quality assessment of total RNA’s quality was performed trough capillary electrophoresis with Fragment Analyzer system (Agilent Technologies) with RNA Kit (15nt) (Agilent Technologies). RNA libraries were prepared with Truseq RNA Exome (Illumina) performing enrichment with Illumina Exome Panel—Enrichment Oligos Only, following the protocol provided by the vendor. Paired end sequencing (75 × 75) was performed with a NextSeq 500 (Illumina).

### Bioinformatic analysis

Bioinformatic analysis of sequencing data was performed with in-house pipelines using the Snakemake [56] workflow management system for process optimization [57]. In both cases, after demultiplexing, FASTQs quality was checked with FASTQC [58] and trimming was performed with Trimmomatic [59]. For DNA target sequencing data, FASTQ files were mapped to the reference genome (GRCh37/hg19) with the Burrows-Wheeler Aligner [60] using bwa-*mem* algorithm. Sorting, PCR duplicates marking and depth of coverage was computed with GATK [61]. Variant calling of Single Nucleotide Polymorphisms (SNPs) and small indels was performed using Strelka2 [62], setting the analysis for germline variant discovery. Variants were then filtered according to quality parameters and constrains provided by the genomic regions covered by the target sequencing panel. Variant Call Format (VCF) files were annotated with BaseSpace Variant Interpreter (Illumina). Variant discovery was validated on multiple functional loci, such as codon 129 in the *PRNP* gene and/or *APOE* genotype. Missense variants effect prediction was estimated with SIFT [63] and Polyphen2 [64]. RNA sequencing data were mapped to the reference genome (GRCh37) with STAR aligner [65] with default setting and read counts quantification. Aligned bam files are sorted and marked for PCR duplicates with GATK [61]. RNA editing events were assessed with REDItools [66] (with default setting unless stated) in the 16 samples in which both DNA and RNA sequencing data were acquired. Despite it is in principle possible to perform RNA editing calls on RNAseq data alone, we chose a conservative approach limiting the analysis at the 118 genes covered by both DNA target sequencing and RNA sequencing. Using as reference in this part the work of Wu et al. [54] we applied the following-more stringent- quality filters to keep only well supported RNA editing calls: only calls not present in the DNA data were kept and called with a coverage higher than 30, a Q score higher than 30, and a frequency higher than 0.2 in the RNA data were kept, with the aim of removing calls with too few support on the RNA (possibly technical artifacts). Finally, only A-to-G changes, consistent with A-to-I editing that represent the primary types of ADAR-mediated RNA editing, were kept.

### Statistical analysis of genomic variants

The genetic information contained in VCF files was transformed into binary data through an in-house python script (https://github.com/UniboDIFABiophysics/binaryVCF), generating a matrix in which each row represents a variant reported in the provided VCF files at least once (which is encoded as chromosome number-position-reference allele- alternate allele) and each column is named after an ID assigned to each sample. In each cell of the matrix is reported the number of alternative alleles for each locus, thus 0 indicates that the variant is not present in the VCF file of the patient whereas 1 indicates its presence in heterozygosity and 2 the presence of the variant in homozygosity. This matrix was used as input for machine learning methods and statistical analysis, following the workflow recently proposed by Tarozzi et al. [21]. Allele frequencies of each variant described at least in one patient of our cohort were calculated and then compared with those reported in the GnomAD database [67] for the non-Finnish European population using Fisher’s exact test and Benjamini–Hochberg multiple test correction. The same statistical test was used to compare allele frequencies between samples from MM1 and VV2subtypes.

### Machine learning analysis of genomic variants

The overall genomic information of the dataset carried in the ternary matrix was used as input for supervised and unsupervised analysis, using SciKit-Learn [68], Seaborn [69], Plotly express [70], pandas, Numpy [71] and SciPy [72] packages. To visualize such high dimensional data, dimensionality reduction was achieved with Principal component analysis (PCA). SciKit Learn dendrograms and K-Means were used as clustering. As supervised methods, decision trees on binary data labelled accordingly to the different subtypes of each patient were used. The classifier was trained on a random selection of 2/3 of the dataset and adequate branching depth was set to avoid overfitting. The classification rules were tested on a validation set represented by the remaining 1/3 of the dataset. Results are expressed through the parameters Precision, Recall and F1 score. Precision is the ratio of correctly predicted observation to the total predicted positive observations (True Positive/True Positive + False Positive), Recall is the ratio of correctly predicted positive observations to all observations in actual class (True Positive/True Positive + False Negative), F1 Score is the harmonic mean of Precision and Recall (F1 Score = 2 × (Recall × Precision)/(Recall + Precision)).

### Differential gene expression analysis

Differential gene expression (DGE) was computed on read counts files output of STAR, using DeSeq2 [73]. Based on the gene-level QC performed with default settings, sex, experimental batch, disease duration and post-mortem conservation values were used as parameters of internal sources of variation. Shrinkage parameters of log2FoldChange were estimated with “apeglm” [74] method on the comparison between VV2 and MM1. Significance cut-off of p < 0.05 was used and multiple test correction was performed with Benjamini-Hochberg. Results of DGE analysis were the annotated using the R package Annotables on human genome GRCh37. Validation was performed with digital droplet PCR (Additional File 1, “RNAseq Validation: Digital Droplet PCR”).

### Functional enrichment analysis

Functional enrichment analysis was performed with over-representation analysis (ORA) and Functional Class Scoring (FCS) approaches. Functional analysis with both approaches was performed using the Bioconductor packages “ClusterProfiler” [75], “g:profiler” [76], “DOSE” [77] and “Pathview” [78] on Gene Ontology [79] and KEGG [80] databases. Representation of enriched over and under expressed pathways was performed using EnrichmentMap and Annotables on Cytoscape [81]. To further explore the biological interplay of differentially expressed genes, we performed protein–protein interaction analysis (PPI) mapping under and over expressed genes on STRING interactome [82], considering only physical interactions. Results were functionally interpreted with over representation analysis using KEGG database to discover pathways significantly enriched based on the physical interactome.

## Supplementary Information

**Additional file1**. **Table S1**: Clinical information on the dataset used in the genomic layer. For each sample, are reported subtype, disease duration expressed in months, age of onset of the disease (years), markers of co-pathology and biological sex. Males and females were equally distributed in the cohort and in the two subgroups, the average age of onset in the MM1 group was 65.8 years (σ = 7.6 years) and in the VV2 was 65.7 (σ = 8.5 years). Disease duration in the MM1 group was 3.1 (σ = 1.8 months) and in the VV2 6.8 months (σ = 2.4 months). **Table S2**: most relevant results of the functional analysis performed on the 36 genes harboring at least one variant with a significantly different allele frequency in the sCJD cohort compared to the healthy European population. Legend: GO:BP= Gene Ontology Biological Processes, GO:CC = Gene Ontology Cellular Component, WP = Wiki Pathway database. **Table S3**: Sites in which RNA editing events were observed in this study. “Chromosome” and “Position” define the genomic locus in which the editing event was observed, “Consequence” the predicted functional change. **Table S4**: Top results of functional enrichment analysis of genes involved in RNA editing modifications in the full cohort. **Table S5**: Top biological processes over expressed according to over-representation analysis of differentially expressed genes in the MM1 compared to VV2 samples. **Table S6**: Top fifteen most statistically significant differentially overexpressed genes in the VV2 subtype compared to MM1, according to RNA sequencing data. **Table S7**: Top fifteen most statistically significant differentially overexpressed genes in the MM1 subtype compared to VV2, according to RNA sequencing data. **Table S8**: Genes analyzed in the target sequencing analysis with the Neurodegeneration panel (Illumina). **Table S9**: Enrichment scores of cell-type enrichment analysis derived from bulk RNA seq. Here are reported enrichment scores for Astrocytes, Endothelial cells, Macrophages, Macrophages M1, Macrophages M2 and Neurons for each sample and the average value per disease subtype.

## Data Availability

The data supporting the conclusions of this article are available in the European Variation Archive - EMBL-EBI (https://www.ebi.ac.uk/eva/) and European Nucleotide Archive - EMBL-EBI (https://www.ebi.ac.uk/ena/). Genomic data (VCF files) are associated with the ID Project: PRJEB57852. FASTQ files of RNAseq experiment are associated with the ID: Project: PRJEB57720.

## Abbreviations

SCJD: Sporadic Creutzfeldt-Jakob disease
PD: Parkinson disease
AD: Alzheimer disease
DGE: Differential gene expression
LFC: Log fold change
DEG: Differentially expressed genes
GO: Gene ontology
GSEA: Gene set enrichment analysis
PCA: Principal component analysis
SNV: Single nucleotide variant
PPI: Protein–Protein interaction
