# Supplementary material for: Genomic, transcriptomic and RNA editing analysis of human MM1 and VV2 sporadic Creutzfeldt-Jakob disease
Source: Acta Neuropathol Commun. 2022 Dec 14;10:181. doi: 10.1186/s40478-022-01483-9 (PMC9749175; doi:10.1186/s40478-022-01483-9)
Supplement: Supplementary file 1 — Additional file1. Table S1: Clinical information on the dataset used in the genomic layer. For each sample, are reported subtype, disease duration expressed in months, age of onset of the disease (years), markers of co-pathology and biological sex. Males and females were equally distributed in the cohort and in the two subgroups, the average age of onset in the MM1 group was 65.8 years (σ = 7.6 years) and in the VV2 was 65.7 (σ = 8.5 years). Disease duration in the MM1 group was 3.1 (σ = 1.8 months) and in the VV2 6.8 months (σ = 2.4 months). Table S2: most relevant results of the functional analysis performed on the 36 genes harboring at least one variant with a significantly different allele frequency in the sCJD cohort compared to the healthy European population. Legend: GO:BP= Gene Ontology Biological Processes, GO:CC = Gene Ontology Cellular Component, WP = Wiki Pathway database. Table S3: Sites in which RNA editing events were observed in this study. “Chromosome” and “Position” define the genomic locus in which the editing event was observed, “Consequence” the predicted functional change. Table S4: Top results of functional enrichment analysis of genes involved in RNA editing modifications in the full cohort. Table S5: Top biological processes over expressed according to over-representation analysis of differentially expressed genes in the MM1 compared to VV2 samples. Table S6: Top fifteen most statistically significant differentially overexpressed genes in the VV2 subtype compared to MM1, according to RNA sequencing data. Table S7: Top fifteen most statistically significant differentially overexpressed genes in the MM1 subtype compared to VV2, according to RNA sequencing data. Table S8: Genes analyzed in the target sequencing analysis with the Neurodegeneration panel (Illumina). Table S9: Enrichment scores of cell-type enrichment analysis derived from bulk RNA seq. Here are reported enrichment scores for Astrocytes, Endothelial cells, Macrophages, Macrophages [file 40478_2022_1483_MOESM1_ESM.docx]

**Supplementary material for:**

**Genomic, transcriptomic and RNA editing analysis of human MM1 and VV2 sporadic Creutzfeldt-Jakob Disease.**

Martina Tarozzi^1^, Simone Baiardi^1,2^, Claudia Sala^1^, Anna Bartoletti-Stella^1^, Piero Parchi^2,3^, Sabina Capellari^2,3^ᵠ*, Gastone Castellani^1^ᵠ.

1 Department of Experimental, Diagnostic and Specialty Medicine, University of Bologna, Italy, 40139 Bologna, Italy.

2 IRCCS Istituto delle Scienze Neurologiche di Bologna, 40139 Bologna, Italia.

3 Department of Biomedical and Neuromotor Sciences, University of Bologna, 40139 Bologna, Italy.

*Corresponding author: Sabina Capellari, sabina.capellari@unibo.it

ᵠ These authors equally contributed to the work

SUPPLEMENTARY RESULTS

| **Sample** | **Strain** | **Disease Duration** | **Age of Onset** | **Co-pathology** | **Sex** | **Post-mortem (h)** |
| --- | --- | --- | --- | --- | --- | --- |
| #1 | VV2 | 13 | 64 | A-beta 0, tau 0 | F | NA |
| #2 | VV2 | 6 | 57 | A-beta 2, tau + | F | 22 |
| #3 | VV2 | 7 | 78 | A-beta 0, tau 1 | M | NA |
| #4 | VV2 | 6 | 49 | A-beta 0, tau 0 | F | NA |
| #5 | VV2 | 5 | 75 | A-beta 3, tau 0 | M | NA |
| #6 | VV2 | 5 | 58 | A-beta 0, tau 0 | F | NA |
| #7 | VV2 | 6 | 79 | A-beta 3, tau 1 | F | NA |
| #8 | VV2 | 6 | 63 | A-beta 0, tau 0 | M | NA |
| #9 | VV2 | 5 | 60 | A-beta 1, tau 1 | M | NA |
| #10 | VV2 | 8 | 61 | A-beta 2, tau 0, CAA | F | NA |
| #11 | VV2 | 10 | 71 | A-beta 3, tau + | F | NA |
| #12 | VV2 | 4 | 78 | A-beta 0, tau II | F | 30 |
| #13 | VV2 | 5 | 49 | A-beta 0, tau 0 | F | 44 |
| #14 | VV2 | 7,5 | 66 | A-beta 0, tau 0 | M | 65 |
| #15 | VV2 | 6 | 74 | A-beta 1, tau 1 | M | 46 |
| #16 | VV2 | 7 | 71 | A-beta 0, tau + | F | NA |
| #17 | VV2 | 9 | 64 | NA | F | 30 |
| #18 | VV2 | 6 | 70 | AGD, A-Beta neg | M | 46 |
| #19 | VV2 | 9 | 65 | A-beta 0, tau 1 | F | NA |
| #20 | VV2 | 5,2 | 72 | A-beta 3, tau + | M | NA |
| #21 | VV2 | 2,6 | 64 | A-beta 0, tau ARTAG in 13 | M | NA |
| #22 | VV2 | *14* | 68 | Neg | M | 84 |
| #23 | VV2 | 5 | 61 | NA | F | NA |
| #24 | VV2 | 7 | 59 | A-beta 0, tau 0 | F | NA |
| #25 | MM1 | 2 | 59 | A-beta 0, tau 0 | F | NA |
| #26 | MM1 | 10 | 70 | A-beta 0, tau 0 | F | NA |
| #27 | MM1 | 9 | 62 | A-beta 0, tau + | F | NA |
| #28 | MM1 | 3 | 72 | A-beta 0, tau 0 | M | NA |
| #29 | MM1 | 2 | 59 | A-beta 0, tau + | M | NA |
| #30 | MM1 | 5,5 | 64 | A-beta 3, tau + | F | NA |
| #31 | MM1 | 1,5 | 69 | A-beta 0, tau + | M | NA |
| #32 | MM1 | 2,5 | 76 | A-beta 1, tau + | M | 32 |
| #33 | MM1 | 4,5 | 68 | A-beta 1, tau + | F | 43 |
| #34 | MM1 | 3 | 62 | A-beta 0, tau 1 | M | NA |
| #35 | MM1 | 1 | 67 | A-beta 0, tau + | M | NA |
| #36 | MM1 | 2 | 66 | A-beta 2, tau 1 | M | 37 |
| #37 | MM1 | 2,5 | 65 | A-beta 0, tau 0 | M | NA |
| #38 | MM1 | 1,5 | 68 | A-beta 1, tau 1 | M | NA |
| #39 | MM1 | 3,5 | 43 | Neg | M | NA |
| #40 | MM1 | 1,5 | 65 | A-beta neg, tau 1 | F | 37 |
| #41 | MM1 | 1,5 | 67 | A-beta 1, tau + | M | NA |
| #42 | MM1 | 1 | 61 | A-beta 2, tau + | M | NA |
| #43 | MM1 | 2 | 74 | A-beta 1, tau + | M | 31 |
| #44 | MM1 | 2,5 | 63 | A-beta neg, tau + | F | 30 |
| #45 | MM1 | 1,5 | 67 | A-Beta 1a, tau+ | M | 12 |
| #46 | MM1 | 3 | 57 | Neg. | M | 14 |
| #47 | MM1 | 3,1 | 74 | A-beta 0, tau 0 | M | NA |
| #48 | MM1 | 5,1 | 80 | A-beta 1a, tau 1 | M | NA |

**Table S1:** Clinical information on the dataset used in the genomic layer. For each sample, are reported strain type, disease duration expressed in months, age of onset of the disease (years), markers of co-pathology and biological sex. Males and females were equally distributed in the cohort and in the two subgroups, the average age of onset in the MM1 group was 65.8 years (σ = 7.6 years) and in the VV2 was 65.7 (σ = 8.5 years). Disease duration in the MM1 group was 3.1 (σ = 1.8 months) and in the VV2 6.8 months (σ = 2.4 months).

| **Source** | **Term_Name** | **Term_ID** | **adj_pvalue** |
| --- | --- | --- | --- |
| GO:BP | negative regulation of cell death | GO:0060548 | 0,00075 |
| GO:BP | synapse organization | GO:0050808 | 0,00079 |
| GO:BP | positive regulation of kinase activity | GO:0033674 | 0,00232 |
| GO:MF | protein kinase binding | GO:0019901 | 0,00841 |
| GO:MF | chaperone binding | GO:0051087 | 0,01398 |
| KEGG | Pathways of neurodegeneration - multiple diseases | KEGG:05022 | 0,00363 |
| WP | Prion disease pathway | WP:WP3995 | 0,02679 |
| GO:CC | axon | GO:0030424 | 0,00002 |
| GO:CC | neuronal cell body | GO:0043025 | 0,00032 |

**Table S2:** most relevant results of the functional analysis performed on the 36 genes harboring at least one variant with a significantly different allele frequency in the sCJD cohort compared to the healthy European population. Legend: GO:BP= Gene Ontology Biological Processes, GO:CC = Gene Ontology Cellular Component, WP = Wiki Pathway database.

| **Chromosome** | **Position** | **Consequence** | **Class_Counts** | **Gene** |
| --- | --- | --- | --- | --- |
| chr19 | 42490154 | splicing, intronic | 1 MM1, 1 VV2 | *ATP1A3* |
| chr11 | 47493737 | splicing, intronic | 1 MM1 | *CELF1* |
| chr1 | 207741245 | synonymous | 1 VV2 | *CR1* |
| chr3 | 184039770 | synonymous | 1 VV2 | *EIF4G1* |
| chr22 | 32871936 | intronic | 3 MM1, 4 VV2 | *FBXO7* |
| chr2 | 233712272 | synonymous | 2 VV2 | *GIGYF2* |
| chr7 | 26236175 | splicing, intronic | 4 MM1, 2 VV2 | *HNRNPA2B1* |
| chr7 | 26236172 | splicing, intronic | 3 MM1, 2 VV2 | *HNRNPA2B1* |
| chr12 | 40671989 | Missense (Ile723Val) | 1 VV2 | *LRRK2* |
| chr11 | 85742663 | splicing, intronic | 1 VV2 | *PICALM* |
| chr8 | 27255263 | synonymous | 1 MM1, 2 VV2 | *PTK2B* |
| chr4 | 90749343 | splicing, intronic | 1 VV2 | *SNCA* |
| chr6 | 170871040 | synonymous | 2 MM1, 4 VV2 | *TBP* |
| chr6 | 170871046 | intronic | 2 MM1, 4 VV2 | *TBP* |
| chr6 | 170871013 | synonymous | 2 MM1, 2 VV2 | *TBP* |
| chr9 | 132576302 | synonymous | 1 MM1 | *TOR1A* |
| chr20 | 57022720 | 3'UTR | 2 VV2 e 2 MM1 | *VAPB* |
| chr15 | 62306194 | splicing, intronic | 6 MM1, 6 VV2 | *VPS13C* |

**Table S3:**  Sites in which RNA editing events were observed in this study. “Chromosome” and “Position” define the genomic locus in which the editing event was observed, “Consequence” the predicted functional change.

| SOURCE | TERM NAME | TERM ID | ADJ PVAL |
| --- | --- | --- | --- |
| GO:MF | SNARE binding | GO:0000149 | 0,02082 |
| GO:BP | synaptic vesicle transport | GO:0048489 | 1.719 x10^-7^ |
| GO:BP | synaptic vesicle localization | GO:0097479 | 7.452 x10^-7^ |
| GO:BP | presynaptic endocytosis | GO:0140238 | 1.296x10^-6^ |
| GO:BP | synaptic vesicle endocytosis | GO:0048488 | 1.296x10^-6^ |
| HP | Akinesia | HP:0002304 | 1.372x10^-11^ |
| HP | Bradykinesia | HP:0002067 | 1.487x10^-11^ |
| HP | Parkinsonism | HP:0001300 | 6.237x10^-11^ |
| GO:CC | inclusion body | GO:0016234 | 4.474x10^-5^ |
| GO:CC | neuronal cell body | GO:0043025 | 1.864x10^-4^ |
| GO:CC | distal axon | GO:0150034 | 2.458x10^-4^ |

**Table S4:** Top results of functional enrichment analysis of genes involved in RNA editing modifications in the full cohort.

| **ID** | **Description** | **GeneRatio** | **BgRatio** | **pvalue** | **p.adjust** | **qvalue** |
| --- | --- | --- | --- | --- | --- | --- |
| GO:0043087 | regulation of GTPase activity | 29/469 | 457/19179 | 3.11E+08 | 0.004 | 0.003477 |
| GO:0043547 | positive regulation of GTPase activity | 26/469 | 385/19179 | 3.31E+08 | 0.004 | 0.003477 |
| GO:0016054 | organic acid catabolic process | 22/469 | 294/19179 | 3.71E+08 | 0.004 | 0.003477 |
| GO:0046395 | carboxylic acid catabolic process | 22/469 | 294/19179 | 3.71E+08 | 0.004 | 0.003477 |
| GO:0046322 | negative regulation of fatty acid oxidation | 5/469 | 12/19179 | 5.88E+08 | 0.005 | 0.004414 |
| GO:0008360 | regulation of cell shape | 15/469 | 160/19179 | 9.37E+08 | 0.006 | 0.005773 |
| GO:0051056 | regulation of small GTPase mediated signal transduction | 23/469 | 338/19179 | 1.08E+09 | 0.006 | 0.005773 |
| GO:0007265 | Ras protein signal transduction | 24/469 | 366/19179 | 1.29E+09 | 0.007 | 0.006045 |
| GO:0040001 | establishment of mitotic spindle localization | 7/469 | 35/19179 | 1.86E+09 | 0.007 | 0.006771 |
| GO:0030198 | extracellular matrix organization | 26/469 | 425/19179 | 1.91E+09 | 0.007 | 0.006771 |

**Table S5:** Top biological processes over expressed according to over-representation analysis of differentially expressed genes in the MM1 compared to VV2 samples.

| **Gene name** | **Protein** | **P_adj** |
| --- | --- | --- |
| UBE2N | Ubiquitin-conjugating enzyme E2 N | 0,00025 |
| OR2M2 | Olfactory receptor 2M2 | 0,00066 |
| TUBA1B | Tubulin alpha-1B chain | 0,00066 |
| RTN4 | Reticulon-4 | 0,00066 |
| SGTB | Small glutamine-rich tetratricopeptide repeat-containing protein beta | 0,00101 |
| CHN1 | N-chimaerin | 0,00101 |
| EIF4A2 | Eukaryotic initiation factor 4A-II | 0,00101 |
| HMGCR | 3-hydroxy-3-methylglutaryl-coenzyme A reductase | 0,00122 |
| SLC25A14 | Brain mitochondrial carrier protein 1 | 0,00122 |
| PIK3R1 | Receptor tyrosine-protein kinase erbB-4 | 0,00122 |
| UBFD1 | Ubiquitin domain-containing protein UBFD1 | 0,00122 |
| LIPH | Lipase member H | 0,00123 |
| SLC39A10 | Zinc transporter ZIP10 | 0,00123 |
| GNE | Bifunctional UDP-N-acetylglucosamine 2-epimerase/N-acetylmannosamine kinase | 0,00123 |
| HSPH1 | Heat shock protein 105 kDa | 0,00125 |

**Table S6:** Top fifteen most statistically significant differentially overexpressed genes in the VV2 subtype compared to MM1, according to RNA sequencing data.

| **Gene Name** | **Protein** | **P_adj** |
| --- | --- | --- |
| COLCA2 | POU class 2 homeobox associating factor 3 | 0,00046 |
| C9orf43 | Uncharacterized protein C9orf43 | 0,00122 |
| MRC2 | C-type mannose receptor 2 | 0,00123 |
| PAPLN | Papilin | 0,00169 |
| C10orf131 | Protein CC2D2B | 0,00170 |
| PHYHD1 | Phytanoyl-CoA dioxygenase domain-containing protein 1 | 0,00170 |
| C1orf228 | Armadillo-like helical domain containing protein 1 | 0,00196 |
| CDK5RAP3 | CDK5 regulatory subunit-associated protein 3 | 0,00196 |
| CDC14A | Dual specificity protein phosphatase CDC14A | 0,00207 |
| SLC38A10 | Putative sodium-coupled neutral amino acid transporter 10 | 0,00221 |
| NUTM2B | NUT family member 2B | 0,00259 |
| PRODH | Proline dehydrogenase 1, mitochondrial | 0,00281 |
| ALPL | Alkaline phosphatase, tissue-nonspecific isozyme | 0,00318 |
| ARGLU1 | Arginine and glutamate-rich protein 1 | 0,00318 |
| ABCA4 | Retinal-specific phospholipid-transporting ATPase ABCA4 | 0,00323 |
| TTC23 | Tetratricopeptide repeat protein 23 | 0,00323 |

**Table S7:** Top fifteen most statistically significant differentially overexpressed genes in the MM1 subtype compared to VV2, according to RNA sequencing data.

SUPPLEMENTARY METHODS

##### DNA target sequencing gene panel

| **Gene Name** | **Protein Name** |
| --- | --- |
| *ABCA7* | ATP binding cassette subfamily A member 7 |
| *ABI3* | ABI family member 3 |
| *ADAM10* | ADAM metallopeptidase domain 10 |
| *ADORA1* | Adenosine receptor A1 |
| *AKT1* | AKT serine/threonine kinase 1 |
| *ALS2* | Alsin |
| *ANG* | Angiogenin |
| *ANO3* | Anoctamin-3 |
| *APOE* | apolipoprotein E |
| *APP* | amyloid beta precursor protein |
| *APTX* | Aprataxin |
| *ATM* | Serine-protein kinase ATM |
| *ATP13A2* | Polyamine-transporting ATPase 13A2 |
| *ATP1A3* | Sodium/potassium-transporting ATPase subunit alpha-3 |
| *ATP6AP2* | ATPase H+ Transporting Accessory Protein 2 |
| *BIN1* | Bridging integrator 1 |
| *BTNL2* | Butyrophilin Like 2 |
| *C21orf2* | chromosome 9 open reading frame 2 |
| *CASS4* | Cas scaffold protein family member 4 |
| *CD2AP* | CD2 associated protein |
| *CD33* | CD33 molecule |
| *CELF1* | CUGBP Elav-Like Family Member 1 |
| *CHCHD10* | coiled-coil-helix-coiled-coil-helix domain containing 10 |
| *CHCHD2* | Coiled-Coil-Helix-Coiled-Coil-Helix Domain Containing 2 |
| *CHMP2B* | charged multivesicular body protein 2B |
| *CLCN6* | Chloride Voltage-Gated Channel 6 |
| *CLU* | Clusterin |
| *CR1* | Complement C3b/C4b receptor 1 (Knops blood group) |
| *CSF1R* | Colony stimulating factor 1 receptor |
| *CTSC* | Cathepsin C |
| *DCTN1* | Dynactin subunit 1 |
| *DNAJC6* | DnaJ Heat Shock Protein Family Member C6 |
| *DNMT1* | DNA Methyltransferase 1 |
| *EIF4G1* | Eukaryotic Translation Initiation Factor 4 Gamma 1 |
| *ELAVL1* | ELAV like RNA binding protein 1 |
| *EP300* | E1A binding protein p300 |
| *EPHA1* | [EPH receptor A1](https://www.ncbi.nlm.nih.gov/gene/2041) |
| *EPHA4* | EPH receptor A4 |
| *FBXO7* | F-Box Protein 7 |
| *FERMT2* | Fermitin family member 2 |
| *FIG4* | FIG4 phosphoinositide 5-phosphatase |
| *FUS* | FUS RNA binding protein |
| *GAK* | Cyclin G Associated Kinase |
| *GBA* | Glucosylceramidase Beta 1 |
| *GCH1* | GTP Cyclohydrolase 1 |
| *GIGYF2* | GRB10 Interacting GYF Protein 2 |
| *GRN* | Granulin |
| *HNRNPA1* | Heterogeneous Nuclear Ribonucleoprotein A1 |
| *HNRNPA2B1* | Heterogeneous Nuclear Ribonucleoprotein A2/B1 |
| *HTRA2* | HtrA serine peptidase 2 |
| *INPP5D* | Inositol polyphosphate-5-phosphatase D |
| *ITM2B* | integral membrane protein 2B |
| *LMNB1* | Lamin B1 |
| *LRRK2* | Leucine Rich Repeat Kinase 2 |
| *MAPT* | Microtubule associated protein tau |
| *MARK2* | Microtubule affinity regulating kinase 2 |
| *MARK4* | Microtubule affinity regulating kinase 4 |
| *MEF2C* | Myocyte enhancer factor 2C |
| *MS4A4A* | Membrane spanning 4-domains A4A |
| *MS4A6E* | Membrane spanning 4-domains A6E |
| *NEFH* | Neurofilament Heavy Chain |
| *NEK1* | NIMA Related Kinase 1 |
| *NME8* | NME/NM23 family member 8 |
| *NOTCH3* | notch 3 |
| *OPTN* | Optineurin |
| *PANK2* | Pantothenate Kinase 2 |
| *PARK2* | Parkin RBR E3 Ubiquitin Protein Ligase |
| *PARK7* | Parkinsonism Associated Deglycase |
| *PFN1* | Profilin 1 |
| *PICALM* | Phosphatidylinositol binding clathrin assembly protein |
| *PINK1* | PTEN Induced Kinase 1 |
| *PLA2G6* | Phospholipase A2 Group VI |
| *PLCG2* | Phospholipase C gamma 2 |
| *POLG* | DNA Polymerase Gamma, Catalytic Subunit |
| *PRKRA* | Protein Activator Of Interferon Induced Protein Kinase EIF2AK2 |
| *PRNP* | Prion Protein |
| *PRRT2* | Proline Rich Transmembrane Protein 2 |
| *PSEN1* | presenilin 1 |
| *PSEN2* | presenilin 2 |
| *PTK2B* | Protein tyrosine kinase 2 beta |
| *PVRL2* | Nectin Cell Adhesion Molecule 2 |
| *RAB29* | RAB29, Member RAS Oncogene Family |
| *RAB38* | RAB38, Member RAS Oncogene Family |
| *RAB39B* | RAB39B, Member RAS Oncogene Family |
| *RAB7L1* | RAB29, Member RAS Oncogene Family |
| *RIN3* | Ras and Rab interactor 3 |
| *SARM1* | Sterile Alpha And TIR Motif Containing 1 |
| *SCARB2* | Scavenger Receptor Class B Member 2 |
| *SETX* | Senataxin |
| *SGTA* | Small Glutamine Rich Tetratricopeptide Repeat Co-Chaperone Alpha |
| *SLC24A4* | Solute carrier family 24 member 4 |
| *SNCA* | Synuclein Alpha |
| *SOD1* | Superoxide Dismutase 1 |
| *SORL1* | Sortilin related receptor 1 |
| *SPAST* | Spastin |
| *SQSTM1* | sesquestosome 1 |
| *SYNJ1* | Synaptojanin 1 |
| *TAF1* | TATA-Box Binding Protein Associated Factor 1 |
| *TARDBP* | TAR DNA binding protein |
| *TBK1* | TANK binding kinase 1 |
| *TBP* | TATA-Box Binding Protein |
| *TH* | Tyrosine Hydroxylase |
| *THAP1* | THAP Domain Containing 1 |
| *TMEM106B* | Transmembrane Protein 106B |
| *TMEM230* | Transmembrane Protein 230 |
| *TOMM40* | Translocase of outer mitochondrial membrane 40 |
| *TOR1A* | Torsin Family 1 Member A |
| *TP53INP1* | tumor protein p53 inducible nuclear protein 1 |
| *TREM2* | triggering receptor expressed on myeloid cells 2 |
| *TRIP4* | Thyroid hormone receptor interactor 4 |
| *TUBA4A* | Tubulin Alpha 4a |
| *TYROBP* | TYRO protein tyrosine kinase binding protein |
| *UBQLN2* | Ubiquilin 2 |
| *UNC13A* | Unc-13 Homolog A |
| *VAPB* | VAMP Associated Protein B And C |
| *VCP* | Valosin-containing protein |
| *VPS13C* | Vacuolar Protein Sorting 13 Homolog C |
| *VPS35* | VPS35 Retromer Complex Component |
| *ZCWPW1* | zinc finger CW-type and PWWP domain containing 1 |

**Table S8:** Genes analyzed in the target sequencing analysis with the Neurodegeneration panel (Illumina).

#### RNAseq Validation: Digital Droplet PCR

Differential Gene Expression results obtained by RNAseq were validated using droplet digital polymerase chain reaction (ddPCR). Five genes with the most extreme Log2FoldChange were selected for validation among those differentially expressed: Antileukoproteinase (*SLPI*), Interleukin-1 receptor antagonist protein (*IL1RN*), Cytochrome P450 3A5 (*CYP3A5*), Macrophage mannose receptor 1 (*MRC1*) and Olfactory receptor 2M2 (*OR2M2*). Reverse transcription ware carried out on 1μg of DNAse I-treated total RNA with SuperScript VILO cDNA Synthesis Kit (Thermo Fisher Scientific). Reactions for ddPCR assay were performed using the QX200 Droplet Generator, the QX200 Droplet Reader, the C1000 Touch Thermal Cycler, and the PX1 PCR Plate Sealer (Bio-Rad, Hercules, CA, USA) following the manufacturer’s instructions. Reactions were carried out in triplicate using the ddPCR Supermix for Probes (no dUTP). The cDNA copies/unit were quantified using the QuantaSoft Software (Bio-Rad). The following three reference genes were chosen as housekeeping reference for brain tissue based on available literature research(Rydbirk et al. 2016; Durrenberger et al. 2012): *XPNPEP1* (X-prolyl aminopeptidase P1), considered a gold standard reference gene for RNA expression studies in post-mortem human brain tissue, *UBE2D2* (ubiquitin-conjugating enzyme E2D 2), and *CYC1* (cytochrome c1).

#### Cell-type enrichment analysis

Normalized and annotated RNAseq gene counts were used as input for cell-type enrichment analysis with xCell (Aran, Hu, and Butte 2017). Results express enrichment scores of (range ±1) of different cell types, and the average value per disease subtype. These results do not show any significant enrichment in any of the considered cell types in the two CJD subtypes.

| **Enrichment_scores** |  |  |  |  |  |  |  |  |  |  |  |  |  |  |  |  |  |  |
| --- | --- | --- | --- | --- | --- | --- | --- | --- | --- | --- | --- | --- | --- | --- | --- | --- | --- | --- |
| **Cell_type/sample** | #12 | #13 | #14 | #15 | #17 | #18 | #2 | #22 | Average VV2 | #32 | #33 | #36 | #40 | #43 | #44 | #45 | #46 | Averge MM1 |
| Astrocytes | 0,000 | 0,000 | 0,000 | 0,000 | 0,000 | 0,000 | 0,000 | 0,000 | 0,000 | 0,000 | 0,000 | 0,000 | 0,000 | 0,000 | 0,000 | 0,000 | 0,000 | 0,000 |
| Endothelial cells | 0,003 | 0,067 | 0,038 | 0,003 | 0,000 | 0,009 | 0,006 | 0,075 | 0,025 | 0,093 | 0,000 | 0,000 | 0,002 | 0,030 | 0,012 | 0,014 | 0,033 | 0,023 |
| Macrophages | 0,020 | 0,014 | 0,002 | 0,019 | 0,020 | 0,001 | 0,013 | 0,015 | 0,013 | 0,014 | 0,012 | 0,003 | 0,005 | 0,018 | 0,000 | 0,065 | 0,015 | 0,016 |
| Macrophages M1 | 0,013 | 0,008 | 0,004 | 0,012 | 0,010 | 0,001 | 0,002 | 0,005 | 0,007 | 0,014 | 0,007 | 0,001 | 0,002 | 0,018 | 0,000 | 0,051 | 0,015 | 0,013 |
| Macrophages M2 | 0,004 | 0,003 | 0,000 | 0,009 | 0,011 | 0,000 | 0,008 | 0,001 | 0,005 | 0,000 | 0,007 | 0,001 | 0,003 | 0,001 | 0,001 | 0,015 | 0,000 | 0,004 |
| Neurons | 0,034 | 0,001 | 0,008 | 0,037 | 0,044 | 0,036 | 0,023 | 0,000 | 0,023 | 0,000 | 0,030 | 0,035 | 0,008 | 0,005 | 0,019 | 0,003 | 0,000 | 0,012 |

**Table S9**: Enrichment scores of cell-type enrichment analysis derived from bulk RNA seq. Here are reported enrichment scores for Astrocytes, Endothelial cells, Macrophages, Macrophages M1, Macrophages M2 and Neurons for each sample and the average value per disease subtype.
